# Evaluation of the Tensile Strength of Absorbable and Non-Absorbable Suture Materials Exposed to Different Toothpaste Solutions: An In Vitro Study

**DOI:** 10.3390/ma19040793

**Published:** 2026-02-18

**Authors:** Zeki Besim, Oğuz Buhara

**Affiliations:** Department of Oral and Maxillofacial Surgery, Faculty of Dentistry, Neat East University, Nicosia 99010, Turkey; oguz.buhara@neu.edu.tr

**Keywords:** dentifrice, wound, degradation, knot, tensile test, suture material, oral surgery

## Abstract

This study aimed to evaluate the mechanical stability of suture materials following exposure to different toothpaste formulations during the early stages of wound healing. Absorbable and non-absorbable suture materials were tested and each suture was tied with five knots on silicone rods. Four different toothpastes and artificial saliva (control) were used. Sutures were exposed to the toothpastes at specific times on certain days, and stored in artificial saliva. Artificial saliva was used to simulate the oral environment. The study duration was 14 days. Tensile strength was tested on selected days throughout a 14-day period. All sutures showed a gradual reduction in tensile strength over time. The least tensile strength reduction was in the artificial saliva group, while most suture degradation was seen in whitening toothpaste group, particularly in absorbable sutures. The findings of this study show that chemical components in certain toothpaste formulations may degrade the sutures during healing. Within the limitations of this in vitro study, the use of the whitening toothpaste group during early wound healing may warrant careful consideration in situations where wound stability is critical.

## 1. Introduction

Wound healing is one of the key aims of all surgical procedures and it directly affects both patient comfort and recovery process. Healing is a dynamic biological process that progresses through several stages. Moreover, it is affected by local and systemic factors [1]. There are three types of wound healing; primary, secondary, and tertiary healing. In primary healing, tissues are approximated to restore their original form and regain structural integrity [2,3,4,5]. Sutures play a key role in maintaining stability which bring wound edges together and support healing over time [6]. If a knotted suture fails it may result in further postoperative complications [7].

In the oral environment, the healing differs greatly from other anatomical regions. Constant saliva, amount of vascularization, and movements such as chewing, speaking, and swallowing create a biological environment. It is important to understand how suture materials behave under these conditions [3,7]. In clinical dentistry, sutures play an important role during the early stages of healing. They maintain wound closure, distribute tension, and keep the tissues in place for the wound to regain its sufficient tensile strength [8].

Sutures in wound closure were used for a very long time and historical evidence shows that they were using them in ancient medical practice around 3500 BC [8]. Maintaining intraoral sutures can be difficult because the material is constantly exposed to host tissues, enzymatic activity, saliva, and bacterial biofilms [8,9]. Structural integrity of the suture materials can be influenced by temperature and pH fluctuations from dietary habits. A recent in vitro study reported that beverages such as cola, fruit juices, coffee, and sweetened milk can alter the pH [10]. Because of the liquid nature of fluids, they have less direct contact with oral tissues but their prolonged acidity can still accelerate the degradation of suture materials. Additionally, this leads to early loss of tensile strength, impaired tissue adaptation, and a higher risk of secondary infection [11]. Toothpastes that are routinely used twice daily may interact with intraoral suture materials.

Suture materials can be classified according to their origin (natural or synthetic), structure (monofilament or multifilament), and degradability (absorbable or non-absorbable) [12,13]. In absorbable sutures, Polydioxanone (PDO) is an absorbable synthetic monofilament which offers smooth handling and low bacterial adherence [14]. Polyglactin (PGLA) is another absorbable suture material with braided copolymer that offers faster absorption and protective mechanical properties [15,16]. In contrast, non-absorbable sutures such as silk and polypropylene are often preferred for their long-term stability. Silk remains popular due to its excellent handling characteristics [17]. Polypropylene (PP) has advantages such as low tissue drag, reduced stiffness, minimal reactivity, and stable tensile strength under variable pH conditions [18,19].

The aim of this in vitro study was to evaluate how different toothpaste formulations affect the tensile strength of various absorbable and non-absorbable suture materials. It is important to clarify the clinical relevance of suture materials in postoperative care. The null hypotheses were as follows:

1. The type of suture material would not significantly affect tensile strength.
2. Exposure to different toothpastes would not significantly affect the tensile strength of the suture materials.
3. Time would not significantly affect the tensile strength of the suture materials.
4. The interaction between suture material, toothpaste and progression of time would not affect the tensile strength of the material.

## 2. Materials and Methods

This in vitro study was carried out at the Research Laboratory of the Faculty of Dentistry, Near East University, between March and June 2025. Four different suture materials were tested, including two non-absorbable (silk and polypropylene) and two absorbable types (polyglactin 910 [PGLA] and polydioxanone [PDO]).

A total of 600 specimens were prepared, with 150 samples for each suture material. Each specimen represented a single suture segment prepared for tensile strength testing. Since tensile strength testing is a destructive method, each specimen was tested only once, and independent specimens were used for each immersion group and each evaluation time point (baseline, days 3, 7, and 14). These were divided into five subgroups (*n* = 30 per group) based on the immersion medium used: artificial saliva (control), conventional toothpaste, herbal toothpaste, sensitivity toothpaste, and whitening toothpaste. These toothpaste categories were selected because they represent the most commonly used oral hygiene products in daily clinical practice. Conventional toothpastes are routinely used by the general population, whitening toothpastes are frequently preferred by patients with esthetic concerns, sensitivity toothpastes are commonly recommended for patients experiencing dentin hypersensitivity, particularly in the postoperative period, and herbal toothpastes are often chosen by patients who prefer natural formulations for daily oral care.

The minimum required sample size was calculated using G*Power software (Version 3.1.9.7; Heinrich Heine University, Düsseldorf, Germany). Based on an assumed effect size of 0.25 (Cohen’s f), a power of 85%, and a significance level of 0.05, the analysis indicated that a minimum of 15 specimens per group would be sufficient to detect significant differences.

Artificial saliva (Testonic, Çetin Kimya, İstanbul, Turkey) served as the control medium. The solution was supplied ready to use in accordance with the DIN 53160-1 standard [20] and was maintained in an incubator (Mega-Term E420P Dry Air Sterilizer, Mega-Term, Istanbul, Turkey) at 37 °C during the study period. Four commercially available toothpastes were selected as immersion media:

Conventional Toothpaste: Colgate Maximum Cavity Protection Fluoride Toothpaste (Colgate-Palmolive Company, New York, NY, USA).

Herbal Toothpaste: Toothpaste with Kamchatka Volcanic Salt (Recipes of Babushka Agafia, Natura Siberica Ltd., Moscow, Russia).

Sensitivity Toothpaste: Colgate Sensitive Instant Relief Repair + Gum Care (Colgate-Palmolive Company, New York, NY, USA).

Whitening Toothpaste: Signal White Now (Unilever, Rotterdam, The Netherlands).

The key ingredients of the toothpaste slurries were documented, and pH values were measured using a digital pH meter (SevenEasy™ pH meter, Mettler-Toledo GmbH, Greifensee, Switzerland); the corresponding data are presented in Supplementary Table S1.

Each toothpaste group was assigned a separate soft-bristled toothbrush. Brushing was performed twice daily by a single operator (Z.B.) using standardized finger pressure. Before each use, the toothbrush was moistened slightly with water, and a pea-sized amount of toothpaste according to the manufacturer’s recommendation was applied to the sutures. Each brushing session lasted five minutes to standardize mechanical exposure across specimens. The control group was subjected to the same standardized brushing protocol using a soft-bristled toothbrush moistened saline, without the application of toothpaste, to ensure that mechanical brushing effects were consistent across all groups. After brushing, the sutures were rinsed with saline and placed back into artificial saliva. Before mechanical testing, the sutures were gently removed from artificial saliva and immediately fixed in the testing machine without any drying procedure. All specimens were stored in an incubator at 37 °C throughout the experiment. Tensile strength testing was performed at baseline (day 0) and on days 3, 7, and 14.

**Figure 1:**
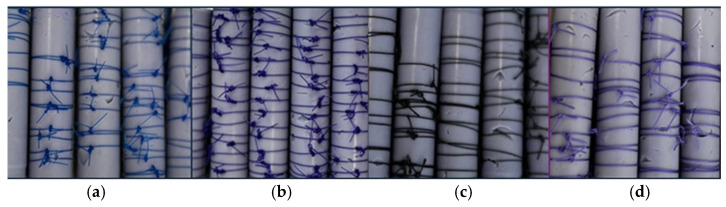
Representative images of sutures placed on elastomeric rods. (**a**) Polypropylene (blue), (**b**) Polydioxanone (violet) (**c**) Silk (black), and (**d**) Polyglactin (light purple).

Polyvinyl siloxane impression material (Variotime^®^ Handmix Easy Putty Refill, Kulzer GmbH, Hanau, Germany) was injected into 5 mL syringe barrels and allowed to polymerize. Once set, the material was removed to create standardized cylindrical molds. Simple interrupted sutures were tied onto each elastomeric cylinder using a single square knot with five throws. Approximately 15 sutures were placed per rod, leaving 5 mm between knots to prevent mechanical interference. In total, 600 suture specimens were distributed across 40 rods, all samples were stored in artificial saliva until testing. Representative images of the sutures placed on elastomeric rods are shown in Figure 1.

**Figure 2:**
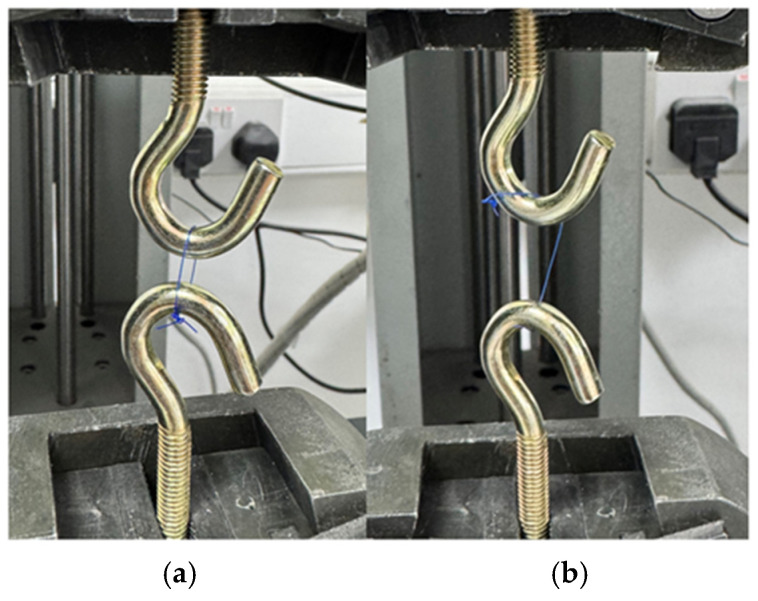
(**a**) Suture fixing on the testing hooks. (**b**) Suture appearance after tensile fracture.

Tensile strength was measured on several days (0, 3, 7, and 14 days) using a Marestech Universal Testing Machine (Mares Engineering, İstanbul, Turkey). The sutures were fixed between two opposing metal grips and subjected to uniaxial tensile loading at a constant crosshead speed of 10 mm/min until failure. The maximum force recorded at rupture was expressed in Newtons (N). The fixation of the sutures on the testing hooks and their appearance after tensile fracture are shown in Figure 2.

All data were analyzed using mean ± standard deviation (SD). The Kolmogorov–Smirnov test was applied to verify the normality of data distribution. A repeated-measures three-way ANOVA was used to analyze the effects of suture type, immersion medium, and time, and the interactions. When significant effects were found, Bonferroni post hoc tests were conducted for pairwise comparisons. A significance level of *p* < 0.05 was adopted for all tests. Statistical analyses were carried out using IBM SPSS Statistics for Windows, Version 25.0 (IBM Corp., Armonk, NY, USA).

## 3. Results

### 3.1. Tensile Strength Analysis

As shown in Table 1, a three-way repeated-measures ANOVA revealed a significant main effect of time on tensile strength (*p* < 0.001), as well as significant interaction effects between time and material, time and solution, and time, material, and solution (*p* < 0.001 for all). Between-subjects analyses demonstrated significant main effects of material and solution, along with a significant material-solution interaction (*p* < 0.001), indicating that tensile strength changes were dependent on both the type of suture material and the solution over time.

The tensile strength values of the suture materials are presented in Table 2 according to solution type and evaluation period (Day 0, Day 3, Day 7, and Day 14). Statistically significant differences were observed both over time and among solutions and materials (*p* < 0.05).

Graphical representations of the tensile strength values presented in Table 2 are provided in Supplementary Figures S1–S4.

### 3.2. Effect of Time in Tensile Strength

Within the same solution and material, significant changes in tensile strength over time were detected. It is indicated by different lowercase superscript letters. Polydioxanone exhibited a significant reduction in tensile strength from Day 0 to Day 14 across all solutions (*p* < 0.05). Among solutions, the most pronounced reduction occurred in the sensitivity toothpaste group. Accordingly, values at Day 14 were significantly lower than at earlier time points. In contrast, polypropylene demonstrated no significant differences among days within the same solution. Thus, it maintained stable tensile strength values during the experimental period (*p* > 0.05). Silk sutures showed a significant time-dependent reduction in tensile strength. This decrease was particularly evident in the Artificial Saliva and Whitening toothpaste solutions. Day 14 values were significantly lower than Day 0 values (*p* < 0.05). In contrast, PGLA protected its tensile strength during the early evaluation periods. However, significant reductions were observed at Day 14 in several solutions (Conventional and Sensitivity toothpaste groups) (*p* < 0.05).

### 3.3. Effect of Solution in Tensile Strength

Comparisons among solutions within the same day showed statistically significant differences. In Table 2, this is shown by different uppercase superscript letters. Specifically, in the polydioxanone group, these differences became evident from Day 3 onward. At later time points, the Sensitivity toothpaste group consistently showed lower tensile strength values than the other toothpaste formulations (*p* < 0.05). Similarly, silk sutures showed noticeable variation across solutions. These differences were most evident at Day 3 and Day 7. In particular, the whitening toothpaste and artificial saliva groups resulted in lower tensile strength values than the herbal and sensitivity toothpaste groups (*p* < 0.05). In contrast, polypropylene showed no significant differences among solutions at any time point, indicating strong resistance to the tested environments (*p* > 0.05). By comparison, PGLA showed fewer inter-solution differences overall; however, significant differences appeared at Day 7 and Day 14, particularly between the whitening toothpaste group and the remaining solutions (*p* < 0.05).

### 3.4. Comparisons of Materials in Tensile Strength

Significant differences between suture materials tested on the same day were observed. In Table 2, they are indicated by symbols (*p* < 0.05). Overall, on all test days, polydioxanone demonstrated the highest tensile strength values. In contrast, polypropylene consistently exhibited the lowest values. Silk and PGLA showed intermediate tensile strength values. Notably, silk displayed greater variability over time. By Day 14, the tensile strength of Silk in Whitening toothpaste was significantly lower than that of all other materials. Conversely, polydioxanone maintained significantly higher values across all solutions. These findings indicate that the response of suture materials to different oral environments is both material- and time-dependent.

## 4. Discussion

Suture materials play a key role in wound healing and surgical success. An ideal suture material should exhibit high tensile strength, adequate stability, and sufficient flexibility. In the present in vitro study, a significant decrease in tensile strength was observed in all suture materials over time (*p* < 0.001). Notably, pronounced changes in mechanical strength were evident particularly during the early postoperative period (the first 7 days), which represents the most critical time frame clinically in terms of wound stability and suture integrity.

The observed reduction in tensile strength may be attributed to hydrolytic and chemical degradation processes occurring during the 14-day experimental period. This finding is consistent with previous studies reporting time as a determining factor affecting suture durability [16,21]. However, the relatively faster decrease observed in the present study may be associated with continuous exposure to dentifrice formulations, which could accelerate suture weakening under simulated oral hygiene conditions. Based on the statistical analyses, both null hypotheses were rejected, as significant differences were detected among suture material type, time, and toothpaste formulation. Different toothpastes produced a significant effect on the tensile strength of the sutures. The highest degradation of suture material was observed in the whitening toothpaste group. This effect may be related to the presence of abrasive particles (e.g., silica) and other formulation-specific chemical factors commonly reported in whitening toothpaste formulations, which may contribute to surface alterations in polymeric sutures [22]. Artificial saliva maintained the most consistent environment. Herbal and sensitivity toothpastes contributed to moderate effects. These variable results may be associated with differences in toothpaste formulations, including fluoride source and other formulation-specific ingredients. The measured pH values were comparable among the tested toothpastes; therefore, pH was not considered a primary explanatory factor within the scope of this study.

The reduction in tensile strength over time highlights the importance of time as a determining factor affecting suture durability. The findings indicate that suture materials exhibit pronounced changes particularly during the early postoperative period, which is clinically critical for wound stability. The observed time-dependent changes did not follow a uniform pattern across materials, suggesting material-specific degradation mechanisms that may influence early clinical performance. From a clinical perspective, this variability should be considered when selecting suture materials for surgical sites exposed to higher functional stress.

A statistically significant interaction among time, suture material, and solution was observed. This indicates that tensile strength loss is not only time-dependent but is also influenced by the intrinsic properties of the suture material and the chemical exposure conditions associated with the tested solutions [21]. Notably, absorbable sutures weakened more rapidly over time when exposed to whitening toothpaste, whereas non-absorbable polypropylene maintained mechanical stability regardless of solution type. Silk sutures exhibited a significant time-dependent reduction in tensile strength, particularly in whitening toothpaste and artificial saliva. By Day 14, silk in contact with whitening toothpaste showed significantly lower tensile strength compared with all other materials. This finding highlights its susceptibility to degradation under the tested experimental conditions. These findings show that environmental factors may play a major role in determining suture performance. Under certain conditions, this role may be greater than the contribution of material composition [23]. The highest initial tensile strength values among all material-solution combinations were observed in the polydioxanone groups on Day 0 (54.27–54.65 N). Over the 14-day period, polydioxanone showed a notable decrease in tensile strength across all toothpaste formulations. The most pronounced reduction occurred in the sensitivity toothpaste group, where values declined from 53.68 N on Day 0 to 41.73 N on Day 14. In contrast, tensile strength remained relatively stable in artificial saliva, decreasing only from 54.27 N to 46.80 N, similar to Emmanuel [24]’s findings.

Polypropylene demonstrated the lowest initial tensile strength values (35.11–35.75 N) and showed minimal change over time regardless of the solution type. This reflects relatively good mechanical stability over time, despite its low baseline tensile strength, and indicates resistance to degradation under the tested experimental conditions rather than high absolute strength. Silk exhibited moderate tensile strength values, with the most substantial loss observed in the whitening toothpaste group, decreasing from 33.89 N on Day 0 to 22.01 N by Day 14. PGLA showed an initial strength range similar to Silk (36.32–37.98 N). Early-period changes were minimal, and a sharper reduction appeared under whitening toothpaste exposure at Day 14 (29.51 N). Overall, these results indicate that the chemical properties of the experimental groups, in some cases, influence suture strength more strongly than the material itself [23].

In contrast, a recent in vitro study evaluating acidic beverages such as cola and coffee reported the strongest degradative effects on suture materials. In the present study, whitening toothpaste formulations produced comparable, or in some cases even greater tensile strength reduction. Conversely, artificial saliva preserved the mechanical properties of all sutures, which is consistent with the findings of Abullais et al. and Alsarhan et al., who reported improved suture stability under less aggressive exposure conditions [16,25]. However, a comparative study of both beverages and toothpastes is needed to clarify the clinical significance.

A recent in vitro study evaluating the effects of mouth rinses on suture degradation reported findings comparable to those of the present study. When 0.12% chlorhexidine gluconate was used as the immersion medium, non-absorbable sutures maintained their tensile strength throughout the 14-day period, whereas absorbable sutures exhibited a significant reduction over time, particularly poliglecaprone (*p* < 0.001). Similarly, in the present study, PGLA exhibited a progressive reduction in tensile strength across the 14-day interval. These results may be related to hydrolytic degradation, which has been reported as a major mechanism affecting the mechanical stability of biodegradable sutures under moist or chemically active conditions [25].

In this in vitro study, a single square knot with five throws was used for all specimens to ensure standardization. The knot is an important step in suturing [7], as it provides mechanical stability to the closure. It should be noted that different knot types or configurations may influence tensile strength measurements by altering stress distribution along the suture material. However, a single knot type was intentionally selected in the present study to maintain consistency across all experimental groups and to allow reliable comparison of the results.

Overall, this study demonstrated that all suture materials experienced time-dependent tensile strength loss, with whitening toothpaste showing the greatest reduction in tensile strength, while artificial saliva provided the most protective environment. The findings of this study suggest that toothpaste composition should be considered when selecting sutures for post-operative care.

The brushing protocol applied in this study was designed to approximate common postoperative oral hygiene practices. Patients are generally advised to continue gentle toothbrushing twice daily during the early healing period to maintain oral hygiene [26]. Although this standardized in vitro protocol cannot fully replicate individual patient behavior or the complex intraoral environment, it allows a controlled evaluation of the time-dependent effects of toothpaste exposure on suture materials.

The experimental duration of the present study was designed to reflect clinical practice, as intraoral sutures are typically removed within 7–14 days and wound closure is generally achieved during this early postoperative period. Therefore, long-term aging and extended storage protocols were not included, as they were considered to have limited direct clinical relevance for suture materials.

The early wound healing period, particularly the first 7 days, is critical for primary wound healing and wound stability. Premature suture failure during this phase may lead to wound dehiscence, increased susceptibility to infection, delayed healing, and a possible transition toward secondary wound healing, depending on the extent of wound separation [2]. In this study, a reduction in tensile strength was observed in sutures exposed particularly to whitening toothpastes. This reduction may contribute to decreased suture stability during the early healing period and may predispose to potential clinical outcomes such as wound dehiscence, secondary healing, and impairment of the wound healing process [8].

One limitation of this study is its in vitro design, which may not fully replicate the complex oral environment, particularly with respect to enzymatic activity, bacterial biofilm formation, and mechanical forces generated during mastication. In addition, in vivo evaluation of suture tensile strength is inherently limited by ethical and methodological constraints. Accurate tensile strength measurement requires the presence of a standardized knot configuration and controlled loading conditions, which cannot be reliably achieved in the oral environment without premature suture removal. Such procedures would interfere with normal wound healing and compromise patient safety. Nevertheless, the in vitro design allowed standardized and controlled evaluation of time-dependent mechanical changes under reproducible conditions. Although the pH values of the tested toothpastes were measured, they were comparable across groups and were not considered a primary explanatory factor. Future studies should focus on other formulation-related characteristics. With respect to fluoride content, all commercially available toothpaste formulations used in this study contained a standardized fluoride concentration of 1450 ppm, with the exception of the herbal toothpaste, which was fluoride-free. Therefore, fluoride content was not considered a primary differentiating factor among the majority of the tested groups. Although peroxide concentration was not measured in the present study, previous in vitro studies have reported that oxidizing agents such as hydrogen peroxide may adversely affect the tensile strength of absorbable suture materials [27]. Therefore, peroxide-related effects cannot be excluded; however, no mechanistic conclusions were drawn within the scope of this study. Important findings have been reported regarding dentin abrasivity [28]. However, as no studies have evaluated the effects of toothpaste abrasivity on suture materials, this aspect could not be assessed within the scope of the present study and should be addressed in future investigations. Future in vivo studies are warranted to further assess suture behavior under clinical conditions using alternative outcome measures.

## 5. Conclusions

This study demonstrated that exposure to different toothpastes caused a reduction in the tensile strength of suture materials. Among the tested materials, polypropylene, as a non-absorbable suture, maintained stable mechanical performance over time. Polydioxanone showed the highest relative resistance to degradation compared with the other suture materials, despite exhibiting measurable tensile strength loss in some toothpaste groups. Whitening toothpaste caused the most aggressive decrease in tensile strength in all suture types. Whitening and sensitivity toothpaste formulations were associated with greater tensile strength loss, particularly in silk and PGLA, with the most marked degradation observed at later evaluation periods. Polypropylene consistently showed the lowest values but maintained stable performance over time. Artificial saliva most effectively preserved suture integrity. These findings suggest that certain characteristics commonly reported in whitening toothpaste formulations, such as abrasive particles and other formulation-specific components, may contribute to suture weakening during the early postoperative period. From a clinical perspective, cautious use of whitening toothpaste formulations during the initial healing phase may be considered, particularly within the first two weeks after surgery. The use of non-whitening and milder formulations may help maintain suture stability; however, these considerations should be interpreted in light of the in vitro nature of the present study.

## Figures and Tables

**Table 1:** Three-way repeated measures ANOVA results.

**Within-Subjects Effects**					
**Cases**	**Sum of Squares**	**df**	**Mean Square**	**F**	***p***
Time	15,410.93 ^a^	3 ^a^	15,410.93 ^a^	386.06 ^a^	<0.001 ^a^
Time*Material	3212.01 ^a^	9 ^a^	356.89 ^a^	26.82 ^a^	<0.001 ^a^
Time*Solution	1012.90 ^a^	12 ^a^	84.41 ^a^	6.34 ^a^	<0.001 ^a^
Time*Material*Solution	3222.41 ^a^	36 ^a^	89.51 ^a^	6.73 ^a^	<0.001 ^a^
Residuals	23,152.74	1740	13.31		
**Between-Subjects Effects**					
**Cases**	**Sum of Squares**	**df**	**Mean Square**	**F**	***p***
Material	120,175.60	3	40,058.53	2662.16	<0.001
Solution	1024.27	4	256.07	44.84	<0.001
Material*Solution	2307.70	12	192.31	30.14	<0.001
Residuals	8727.47	580	15.05		

Note: Type III sum of squares. ^a^ Mauchly’s test of sphericity indicates that the assumption of sphericity is violated (*p* < 0.05). * indicates the interaction effect between the analyzed factors (suture type, immersion medium, and time) in the three-way ANOVA.

**Table 2:** Descriptive statistics for tensile strength (N) with pairwise comparisons.

Material	Day 0	Solution	Day 3	Day 7	Day 14
Polydioxanone	54 ± 2.6 ^b,A,⊛^	Artificial Saliva	49 ± 4.4 ^a,A,⊛^	48 ± 2.1 ^a,A,⊛^	47 ± 2.3 ^a,A,⊛^
	54 ± 2.3 ^c,A,⊛^	Conventional TP	52 ± 3.8 ^b,B,⊛^	51 ± 3.3 ^b,B,⊛^	46 ± 2.2 ^a,A,⊛^
	54 ± 1.0 ^c,A,⊛^	Herbal TP	53 ± 1.1 ^b,B,⊛^	48 ± 0.2 ^b,A,⊛^	46 ± 0.9 ^a,A,⊛^
	55 ± 16.1 ^d,A,⊛^	Sensitivity TP	54 ± 3.3 ^c,B,⊛^	47 ± 2.0 ^b,A,⊛^	42 ± 2.0 ^a,B,⊛^
	54 ± 16.2 ^b,A,⊛^	Whitening TP	48 ± 3.0 ^a,A,⊛^	47 ± 3.0 ^a,A,⊛^	45 ± 10.2 ^a,A,⊛^
Polypropylene	36 ± 1.3 ^a,A,⊕^	Artificial Saliva	35 ± 1.0 ^a,A,⊕^	35 ± 0.9 ^a,A,⊕^	33 ± 0.8 ^a,A,⊕^
	35 ± 7.9 ^a,A,⊕^	Conventional TP	33 ± 1.1 ^a,A,⊕^	33 ± 0.8 ^a,A,⊕^	31 ± 0.6 ^a,A,⊕^
	36 ± 7.9 ^a,A,⊕^	Herbal TP	33 ± 1.0 ^a,A,⊕^	32 ± 1.5 ^a,A,⊕^	32 ± 2.3 ^a,A,⊕^
	35 ± 1.0 ^a,A,⊕^	Sensitivity TP	34 ± 3.3 ^a,A,⊕^	33 ± 1.6 ^a,A,⊕^	31 ± 4.5 ^a,A,⊕^
	35 ± 7.9 ^a,A,⊕^	Whitening TP	35 ± 2.7 ^a,A,⊕^	33 ± 1.9 ^a,A,⊕^	31 ± 3.6 ^a,A,⊕^
Silk	38 ± 1.1 ^c,A,⊕^	Artificial Saliva	28 ± 3.1 ^b,A,⊚^	28 ± 2.6 ^b,A,⊚^	24 ± 0.7 ^a,A,⊚^
	38 ± 0.5 ^b,A,⊕^	Conventional TP	33 ± 0.5 ^b,B,⊚^	28 ± 1.4 ^a,A,⊚^	26 ± 0.7 ^a,A,⊚^
	37 ± 1.3 ^c,A,⊕^	Herbal TP	35 ± 0.9 ^b,B,⊕^	33 ± 0.5 ^b,B,⊕^	30 ± 0.5 ^a,B,⊕^
	37 ± 1.4 ^b,A,⊕^	Sensitivity TP	34 ± 1.5 ^a,B,⊕^	34± 2.7 ^a,B,⊕^	33 ± 0.4 ^a,B,⊕^
	38 ± 0.9 ^d,A,⊕^	Whitening TP	34 ± 0.8 ^c,B,⊕^	26 ± 0.9 ^b,A,⊚^	22 ± 1.4 ^a,A,⊚^
Polyglactin	38 ±1.2 ^a,A,⊕^	Artificial Saliva	37 ± 5.4 ^a,A,⊕^	36 ± 3.8 ^a,A,⊕^	34 ± 2.2 ^a,B,⊕^
	37 ± 0.9 ^a,A,⊕^	Conventional TP	37 ± 0.9 ^a,A,⊜^	34 ± 0.8 ^a,A,⊕^	33 ± 0.7 ^a,B,⊕^
	37 ± 1.0 ^a,A,⊕^	Herbal TP	37 ± 1.2 ^a,A,⊜^	36 ± 0.7 ^a,A,⊚^	35 ± 0.6 ^a,B,⊜^
	37 ± 1.1 ^a,A,⊕^	Sensitivity TP	36 ± 0.7 ^a,A,⊕^	36 ± 0.6 ^a,A,⊚^	35 ± 0.6 ^a,B,⊜^
	37 ± 1.0 ^b,A,⊕^	Whitening TP	37 ± 0.7 ^b,A,⊜^	35 ± 0.9 ^ab,A,⊜^	30 ± 0.6 ^a,A,⊕^

Different superscript lowercase letters indicate significant differences among days in the same row, Different superscript uppercase letters indicate significant differences among solutions in the same column, Different superscript symbols indicate significant differences among materials on the same day.

## Data Availability

The original contributions presented in this study are included in the article/Supplementary Material. Further inquiries can be directed to the corresponding author.

## Supplementary Materials

The following supporting information can be downloaded at: https://www.mdpi.com/article/10.3390/ma19040793/s1, Table S1: Characteristics of the tested toothpastes; Figure S1: Time-dependent tensile strength changes of polydioxanone sutures in artificial saliva and different toothpaste groups; Figure S2: Time-dependent tensile strength changes of polypropylene sutures in artificial saliva and different toothpaste groups; Figure S3: Time-dependent tensile strength changes of polyglactin sutures in artificial saliva and different toothpaste groups; Figure S4: Time-dependent tensile strength changes of silk sutures in artificial saliva and different toothpaste groups.

## Abbreviations

The following abbreviations are used in this manuscript:

PP	Polypropylene
PGLA	Polyglactin 910
PDO	Polydioxanone
TP	Toothpaste
N	Newton
