# Supplementary material for: Evaluation of the Tensile Strength of Absorbable and Non-Absorbable Suture Materials Exposed to Different Toothpaste Solutions: An In Vitro Study
Source: Materials (Basel). 2026 Feb 18;19(4):793. doi: 10.3390/ma19040793 (PMC12941980; doi:10.3390/ma19040793)
Supplement: Supplementary file 1 [file materials-19-00793-s001.zip › materials-4084180-supplementary.pdf]

Table S1. Characteristics of the tested toothpastes.

Toothpaste	Manufacturer	Intended use	Key Ingredients	Whitening agent	Fluoride source	pH value
Colgate Maximum Cavity Protection	Colgate-Palmolive	Conventional	Dicalcium phosphate dihydrate, Hydrated Silica	-	Sodium Monofluorophosphate	6.56
Signal White Now	Unilever	Whitening	Hydrated silica, Optical colorants (CI 74160, CI 74260)	Hydrated silica	Sodium fluoride	6.60
Colgate Sensitive Pro-Relief	Colgate-Palmolive	Sensitivity	Arginine	-	Sodium monofluorophosphate	6.62
Natural Certified Toothpaste Volcanic Salt of Kamchatka	Natura Siberica	Herbal	Volcanic powder,<br>Sea salt,<br>Active charcoal	-	-	6.62

Ingredient information was obtained from official manufacturer websites. The pH values were experimentally measured using a calibrated pH meter.

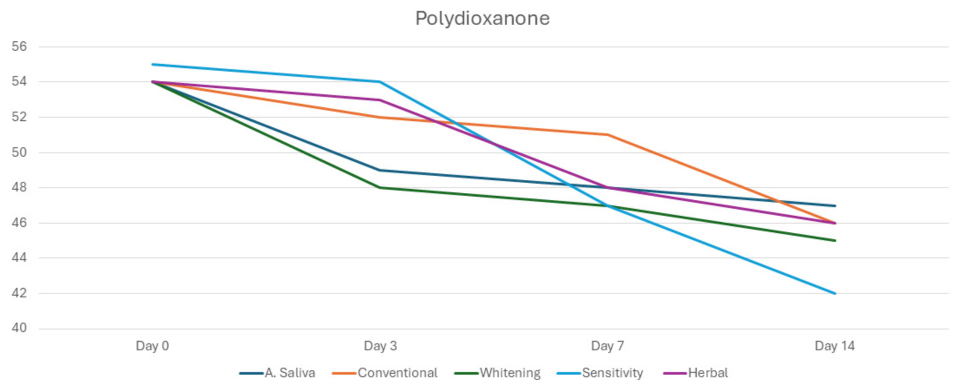

Figure S1. Time-dependent tensile strength changes of polydioxanone sutures in artificial saliva and different toothpaste groups.

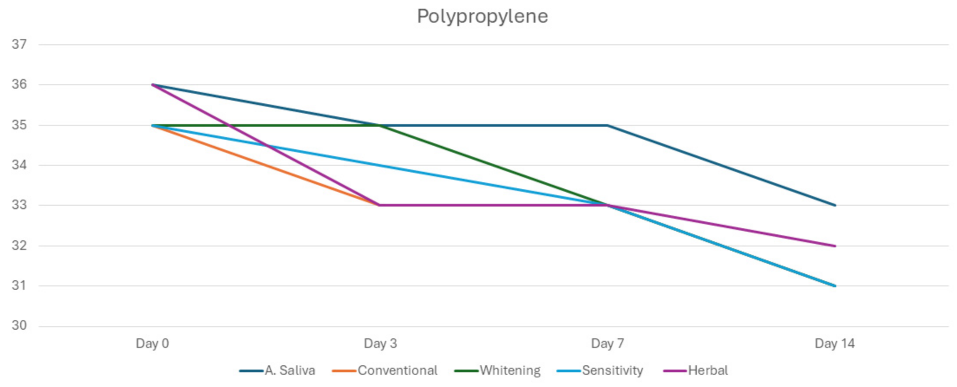

Figure S2. Time-dependent tensile strength changes of polypropylene sutures in artificial saliva and different toothpaste groups.

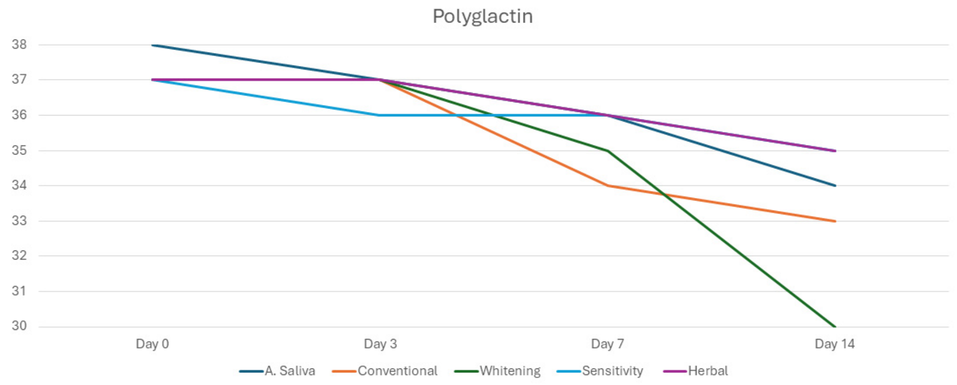

Figure S3. Time-dependent tensile strength changes of polyglactin sutures in artificial saliva and different toothpaste groups.

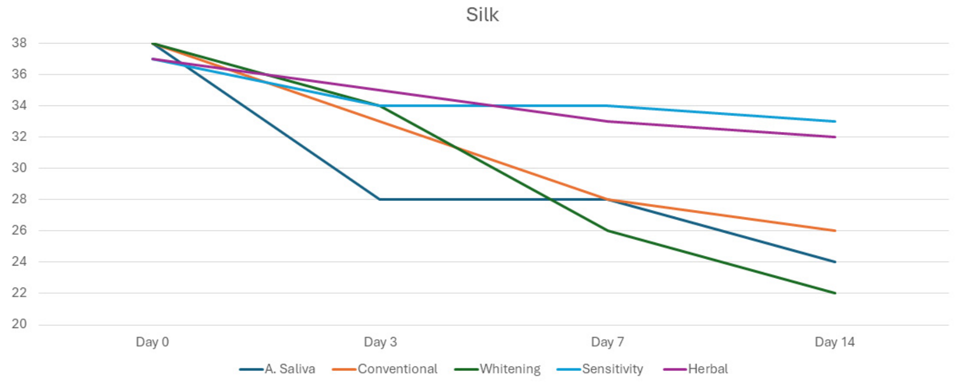

Figure S4. Time-dependent tensile strength changes of silk sutures in artificial saliva and different toothpaste groups.
